# An improved compound Poisson model for the number of motif hits in DNA sequences

**DOI:** 10.1093/bioinformatics/btx539

**Published:** 2017-08-28

**Authors:** Wolfgang Kopp, Martin Vingron

**Affiliations:** Computational Molecular Biology, Max Planck Institute for Molecular Genetics, Berlin, Germany

## Abstract

**Motivation:**

Transcription factors play a crucial role in gene regulation by binding to specific regulatory sequences. The sequence motifs recognized by a transcription factor can be described in terms of position frequency matrices. When scanning a sequence for matches to a position frequency matrix, one needs to determine a cut-off, which then in turn results in a certain number of hits. In this paper we describe how to compute the distribution of match scores and of the number of motif hits, which are the prerequisites to perform motif hit enrichment analysis.

**Results:**

We put forward an improved compound Poisson model that supports general order-*d* Markov background models and which computes the number of motif-hits more accurately than earlier models. We compared the accuracy of the improved compound Poisson model with previously proposed models across a range of parameters and motifs, demonstrating the improvement. The importance of the order-*d* model is supported in a case study using CpG-island sequences.

**Availability and implementation:**

The method is available as a Bioconductor package named ’*motifcounter*’ https://bioconductor.org/packages/motifcounter.

**Supplementary information:**

Supplementary data are available at *Bioinformatics* online.

## 1 Introduction

Transcription factors (TFs) play an essential role in the regulation of gene expression. They function by binding to short sequences known as transcription factor binding sites (TFBSs) which are typically located in promoter or enhancer regions (Alberts *et al.*, 2002). Based on the motif-descriptions of the TFBSs many programs search for occurrences of the motif in a sequence. Since the motifs typically lack specificity, the need arises to determine the statistical significance of a motif match and to delineate how many matches of a motif one would expect to find in a sequence by chance. Relative to this information, TFBSs enrichment can subsequently be inferred for the sequences of interest, e.g. a set of promoters (Pape *et al.*, 2008; Thomas-Chollier *et al.*, 2008).

The binding motif of a TF is typically described either as a consensus sequence or as a position frequency matrices (PFMs) (Stormo, 2000). A PFM tabulates the frequency at which a certain base has been observed at a position of a transcription factor binding site. The logo depiction of a PFM is a common tool of visualizing a TF motif (Schneider and Stephens, 1990). PFMs for many well studied TFs have been collected in different databases, including Transfac (Wingender *et al.*, 1996), Jaspar (Sandelin *et al.*, 2004) or Hocomoco (Kulakovskiy *et al.*, 2013). Many programs are available to scan a sequence with a PFM (Bailey *et al.*, 2009; Chen *et al.*, 1995; Cartharius *et al.*, 2005; Thomas-Chollier *et al.*, 2008). These program are also at the core of the motif enrichment approach, where a set of sequences is scanned for motifs which in those sequences are found more often than expected by chance (Frith *et al.*, 2004; McLeay and Bailey, 2010; Roider *et al.*, 2009; Zambelli *et al.*, 2009).

Different applications require different statistical considerations. When searching for a cutoff for the best matching hits of a PFM in a sequence, one needs to determine the distribution of the corresponding match score. Once a threshold is chosen, one can count the number of different hits a PFM has in a sequence and determine the distribution of this statistic. For consensus strings rather than PFMs this problem has been studied in depth (for review see Reinert *et al.*, 2000). Although practically of considerable importance, the problem of determining the distribution of the number of PFM hits has found less attention (Pape *et al.*, 2008; Rahmann *et al.*, 2003).

Motif search is often employed in regulatory regions which may be made up of CpG-islands, which display dependence between adjacent nucleotides. Such dependencies are frequently ignored by existing methods, including our own earlier method (Pape *et al.*, 2008), which is restricted to using an order-0, or i.i.d., background model. However, CpG-islands can only be adequately modeled by at least an order-1 Markov model which motivates the use of a higher-order background model (Thomas-Chollier *et al.*, 2008).

A further difficulty that we attend to in this work is the overlap structure of PFMs. Clearly, when a motif is repetitive, observing the motif once makes it more likely to find it again, with overlap to the first occurrence. Such a combined occurrence is called a clump. While our earlier work put forward an efficient approximation to compute the statistics for the clumps, we here report an improved way of computing these probabilities. This allows more accurate estimation of the distribution of the number of motif hits, a prerequisite, e.g. for accurate motif enrichment analysis. All these computations will be done for motif hits on the forward and the reverse DNA strand, since in practice one has no prior knowledge where to expect a hit.

The workhorse of computing hit occurrence counts is called a compound Poisson model (Reinert *et al.*, 2000; Waterman, 1995). In contrast to assuming that motif occurrences follow a sequence of i.i.d. Bernoulli trails (Rahmann *et al.*, 2003; Thomas-Chollier *et al.*, 2008), the compound Poisson model can capture the self-overlapping structure of motifs. This aspect renders the compound Poisson model applicable for self-overlapping as well as non-self-overlapping motifs. The compound Poisson model can describe the real distribution accurately as long as the occurrence of a motif is rare, as is normally assumed in practical applications anyway. Originally, the compound Poisson model has been adopted for studying word count frequencies and frequencies of sets of words (Reinert *et al.*, 2000; Waterman, 1995). The framework was later adopted by Pape *et al.* (2008) to study motifs that are given by PFMs. While, for word-pattern centered approaches the hit counts distribution may even be determined exactly, they require enumerating a potentially very large set of words that would gives rise to TFBSs (so-called compatible words) which may be too time-consuming to compute (Zhang *et al.*, 2007).

By contrast, the PFM-based approach efficiently bypasses the enumeration by establishing an approximation which makes it useful even if enumerating all compatible words is too time-consuming.

In this paper, we show how to compute the statistics of motif occurrence counts. We use a higher-order background model and shall demonstrate the importance of higher-order background models in a case study in human CpG islands. We introduce a refined approach for determining the clump size distribution, obtaining more accurate results particularly for self-similar and repeat-like motifs. Unlike earlier methods, we account for matches on both strands, including the possible overlap structure of palindromic motifs. We systematically compare our improved compound Poisson model with the previous model (Pape *et al.*, 2008) and with a frequently used binomial model (Rahmann *et al.*, 2003; Thomas-Chollier *et al.*, 2008) across a range of parameter settings and a large set of motifs. We find that generally, the improved compound Poisson model yields at least similar and frequently more accurate results compared to the other two models, as long as the ‘rare hit’ assumption is met.

## 2 Materials and methods

### 2.1 Motifs, background, motif score and motif hits

Let A={A,C,G,T} denote the alphabet of DNA letters and $w=w_{1}w_{2}\cdots w_{N}$ a sequence of length *N* from this alphabet. The probability of **w** is given by a homogeneous order-*d* Markov model (the *background model*), whose transition probabilities are denoted by $\pi (w_{i-d}\cdots w_{i-1};w_{i})=P(w_{i}|w_{i-1}\cdots w_{i-d})$ and whose stationary distribution is denoted by *μ*. In the case *d* = 0, we set μ=π. Thus, we have

$$P_{B}(w)=\mu (w_{1}\cdots w_{d})\prod_{i=d+1}^{N}\pi (w_{i-d}\cdots w_{i-1};w_{i}).$$

The transition probabilities $\pi (a_{0}\cdots a_{d-1};a_{d})$ are estimated via the maximum likelihood procedure described in (Reinert *et al.*, 2000)

(1) $$\hat{\pi }(a_{0}\cdots a_{d-1};a_{d})=\frac{N(a_{0}\cdots a_{d-1},a_{d})}{\sum_{a_{d}}N(a_{0}\cdots a_{d-1},a_{d})}$$

with N(a) denoting the count of $a\in A^{d+1}$ in $w\in A^{N}$ and under the additional constraints that each word occurs equally likely on both DNA strands and with reversed nucleotide order (from 5′ to 3′ and 3′ to 5′). Those constraints are required since both DNA strands are scanned for motif matches and they are enforced by utilizing the detailed balance condition (see Supplementary Notes Equation (1)–(3)). They also ensure that under the stationary distribution, a word, its reverse complement, and the word with reversed nucleotide order occur equally likely (e.g. μ(AC)=μ(GT)=μ(CA)).

We represent the DNA binding affinity by a position frequency matrix (PFM). A PFM is a |A|×M matrix where |A| denotes the size of the alphabet and *M* denotes the length of the TF binding site. A PFM contains the elements $p_{j}(w)$ which correspond to the frequency of observing nucleotide *w* at position *j*. We shall further assume that all elements of the PFM are strictly positive and its columns are normalized to one such that they represent probabilities. Then, the likelihood of a word $w\prime \in A^{M}$ w.r.t. the PFM is given by

$$P_{M}(w\prime )=\prod_{j=1}^{M}p_{j}(w\prime_{j}).$$

We adopt the commonly used log-likelihood ratio (Li and Tompa, 2006; Rahmann *et al.*, 2003; Touzet *et al.*, 2007), or motif *score*, in order to discriminate likely bound sequences from unbound sequences according to

(2) $$s(w\prime ):=\text{log}\;\left(\frac{P_{M}(w\prime )}{P_{B}(w\prime )}\right)$$

where $w\prime \in A^{M}$ and assume that d≤M for the remainder of this article.

We leverage the motif score in order to determine *motif hits* (or putative TFBSs) by utilizing a pre-determined *score threshold*. Position *i* in a sequence is called a motif hit if $s(w_{i}\ldots w_{i+M-1})$ is greater or equal to the score threshold. According to Neyman and Pearson (1933), it is reasonable to choose a score threshold *t_α_* which is associated with a desired false positive level *α*. Hence, motif hits are called with significance level *α*. In order to choose *t_α_*, we determine the distribution of the scores *P_B_*(*S *=* s*) using an efficient algorithm where we assume the underlying sequence to be generated by an order-*d* background model starting in the stationary distribution *μ* (see Supplementary Notes). A similar approach was reported in the RSAT suite (Thomas-Chollier *et al.*, 2008), although the details of the algorithm were not described there. We obtain the *score threshold t_α_* associated with significance level *α* from *P_B_*(*S *=* s*) by computing $P_{B}(S\geq t_{\alpha })=\alpha$.

For the remainder of this article we omit the subscript *B* as we only focus on distributions that are induced by the background model.

Finally, we define the number of motif hits *X* on both strands of a DNA sequence of length *N* as

$$X=\sum_{i=1}^{N-M+1}Y_{i}+Y\prime_{i}$$

where for convenience we introduced the indicator random variable $Y_{i}:=1[s(w_{i}\cdots w_{i+M-1})\geq t_{\alpha }]$ to reflect TFBS occurrences in random DNA sequences. An additional indicator random variable $Y\prime_{i}$ reflects the corresponding reverse strand event at position *i*.

### 2.2 Compound Poisson distribution

In this section, we recapitulate the compound Poisson model derived by Pape *et al.* (2008).

In the compound Poisson approximation, the distribution of the number of hits is indirectly approximated by modeling the frequency of *clump* occurrences. A clump corresponds to one or more mutually overlapping motif hits. More specifically, a *c-clump* is defined as a clump which contains exactly *c* overlapping motif hits (Reinert *et al.*, 2000). By modeling *c*-*clump* occurrence rates, the compound Poisson approach implicitly accounts for the potentially self-overlapping motif structure.

Formally, the compound Poisson approximation for the number of motif hits is given by

$$X=\sum_{i=1}^{Z}C_{i}$$

where *Z* describes the number of clumps (regardless of how many hits they contain) and ${\left\{C_{i}\right\}}_{1\leq i\leq Z}$ which denote the respective random clump sizes. We assume *Z* to be Poisson-distributed with parameter *λ* and *C_i_* to be i.i.d. random variables. Because, *C_i_* is i.i.d. for all *i*, we shall use *C* to denote the random clump size for any given clump. The probability that any given clump contains *c* motif hits is defined by

(3) $$\theta_{c}:=P(C=c).$$

We defer the derivation of *θ_c_* to Section 2.4.

Importantly, *Z* and *C* are assumed to be independent. Thus, the expected total number of motif hits is given by

(4) $$\begin{array}{c} E[X]=E\left[\sum_{i=1}^{Z}C_{i}\right]=E_{Z}[Z\cdot E_{C}[C]]\\ =E_{Z}[Z]\cdot E_{C}[C]\\ =E_{Z}[Z]\sum_{c>0}c\theta_{c}. \end{array}$$

This expression can also be written as

(5) E[X]=2α(N−M+1)

using the false positive probability *α* for obtaining a hit, the length of the sequence *N*, the motif length *M*, and the factor 2, because the hits are counted on both strands.

Using Equation (4) and (5), the expected number of clump occurrences *λ* is

(6) $$\lambda =E[Z]=\frac{E[X]}{E[C]}=\frac{2\alpha (N-M+1)}{\sum_{c>0}c\theta_{c}}.$$

Finally, employing (Kemp, 1967):

(7) $$P(X=0)=e^{-\lambda }$$

(8) $$P(X=x)=\frac{\lambda }{x}\sum_{x\prime =0}^{x-1}\left(x-x\prime \right)\theta_{x-x\prime }P(X=x\prime )$$

recursively evaluates the compound Poisson approximation, where each time Equation (8) is invoked a clump is added. The summation in Equation (8) then considers all clump sizes.

### 2.3 Self-overlapping hit probabilities

We proceed by deriving the probabilities of obtaining overlapping motif hits, which in turn are necessary for deriving the clump size probabilities *θ_c_*. To this end, we start by explaining *marginal overlapping hit probabilities* from which we subsequently derive the *principal overlapping hit probabilities*. Finally, we consider overlapping hits due to scanning both DNA strands.

#### *2.3.1* Marginal overlapping hit probabilities

Along the line of Pape *et al.* (2008), we shall derive overlapping hit probabilities based on the two-dimensional score distribution P(S=s,S′=s′) where *s* and s′ may correspond to scores at different positions (or strands). Assuming that the background model starts in the stationary distribution *μ*, we propose an extension of the original algorithm that assumes a general order-*d* background model (see Supplementary Notes).

We utilize the algorithm to determine the distribution of the scores at two respective motif start positions 0 and k∈{1,…M−1} simultaneously from which we obtain

(9) $$\gamma_{k}:=P(Y_{k}=1|Y_{0}=1)=\frac{P(S_{k}\geq t_{\alpha },S_{0}\geq t_{\alpha })}{\alpha }.$$

We refer to *γ_k_* as to the *marginal overlapping hit probability* of obtaining an overlapping motif hit *k* positions downstream of a preceding hit $Y_{0}=1$. The adjective ’marginal’ refers to the fact that events in between *Y*_0_ and *Y_k_* (e.g. $Y_{1}Y_{2}\cdots Y_{k-1}$) have been averaged out.

#### *2.3.2* Principal overlapping hit probabilities

In the word-pattern field, *periods* refer to the shifts at which a word potentially overlaps with itself (Reinert *et al.*, 2000; Waterman, 1995). For example, for the word ’AAA’, the periods would be {1, 2}. However, the set of periods potentially explains the overlapping positions redundantly. For example, for ’AAA’, an overlap with period 2 is only possible, if there is a hit at period 1 as well. In other words, period 2, is a consequence of two consecutive hits with period 1. In order to describe overlapping positions non-redundantly, *principal periods* were introduced as such periods that cannot be explained as a mere consequence of another *period* (e.g. as an integer multiple of another period). The only principal period for ’AAA’ equals one.

Motivated by the discussion about *periodicity* (Reinert *et al.*, 2000), we sought to adopt the periodicity concept to PFM-based motifs in order to non-redundantly account for the probability of overlapping motif hits. This can be achieved by excluding intermediate motif hits according to

(10) $$\beta_{k}:=P(Y_{k}=1,Y_{k-1}=0,\ldots Y_{1}=0|Y_{0}=1)$$

for k∈{1,…M−1}. We refer to *β_k_* as to the *principal overlapping hit probability* of obtaining an overlapping hit *k* positions after the event $Y_{0}=1$. A similar approach was also proposed for motifs as generalized strings (Marschall and Rahmann, 2010).

Unfortunately, the exact determination of ${\left\{\beta_{k}\right\}}_{1\leq k<M}$ for general PFMs and an arbitrary score threshold *t_α_* by enumerating DNA words (e.g. compatible words) would require exponential running time (Zhang *et al.*, 2007). However, we propose a novel approximative approach for computing ${\left\{\beta_{k}\right\}}_{1\leq k<M}$ based on ${\left\{\gamma_{k}\right\}}_{1\leq k<M}$ derived above (see Supplementary Notes).

#### *2.3.3* Overlapping hits on both DNA strands

**Fig. 1.**
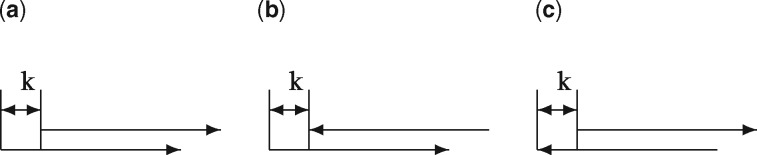
Three types of overlapping hit with a shift of *k* between the motif starts. The arrows pointing to the right and left represent the (5′ → 3′) and (3′ ← 5′) directions of the DNA, respectively

In many applications, we do not know in advance on which DNA strand a TFBS might occur. Therefore, we simply scan both DNA strands for motif hits. However, this might lead to overlapping hits not only on the same strand (as described above), but also on the respective complementary strand. We identified three distinct overlapping hit scenarios: 1) Hits might overlap on the same strand, 2) a forward strand hit $Y_{0}=1$ precedes a reverse strand hit $Y\prime_{k}=1$ and 3) a reverse strand hit $Y\prime_{0}=1$ precedes a forward strand hit *Y _k_* = 1, where *k* denotes the shift between the hits (see Fig. 1). Importantly, we discriminate between cases 2) and 3), because they are not necessarily the same. Case 2) represents a 3′-end overlap of the motif, whereas case 3) represents a 5′-end overlap (see Fig. 1b and c). For example, consider the words ’TCG’ and ’CGT’: ’TCG’ may overlap with its reverse complementary sequence on the 3′-end, but not on the 5′-end, whereas, the opposite is true for ’CGT’.

For the remainder of this article, we consider the events *Y_i_* and $Y\prime_{i}$ in the order $Y_{1}Y\prime_{1}Y_{2}Y\prime_{2}Y_{3}Y\prime_{3}\cdots$ from left to right. This convention ensures that each event is considered exactly once. Note that this also implies that *Y_i_* precedes $Y\prime_{i}$ for all i.

According to the discussion in this section, we extend the *marginal overlapping hit probabilities* to

(11) $$\gamma_{k}:=P(Y_{k}=1|Y_{0}=1)\forall k\in \{1,\ldots M-1\}$$

(12) $$\gamma_{3\prime ,k}:=P(Y\prime_{k}=1|Y_{0}=1)\forall k\in \{0,\ldots M-1\}$$

(13) $$\gamma_{5\prime ,k}:=P(Y_{k}=1|Y\prime_{0}=1)\forall k\in \{1,\ldots M-1\}.$$

They are determined analogously as described above using the two-dimensional score distribution. Depending on the strandedness of the events, the original or the reverse complemented motif is used to determine the scores.

The corresponding *principal overlapping hit probabilities* are given by

(14) $$\beta_{k}:=P(Y_{k}=1,{\left\{Y_{j}=0,Y\prime_{j}=0\right\}}_{1\leq j<k},Y\prime_{0}=0|Y_{0}=1)$$

(15) $$\beta_{3\prime ,k}:=P(Y\prime_{k}=1,{\left\{Y_{j}=0\right\}}_{1\leq j\leq k},{\left\{Y\prime_{j}=0\right\}}_{0\leq j<k}|Y_{0}=1)$$

(16) $$\beta_{3\prime ,0}:=P(Y\prime_{0}=1,|Y_{0}=1)$$

(17) $$\beta_{5\prime ,k}:=P(Y_{k}=1,{\left\{Y_{j}=0,Y\prime_{j}=0\right\}}_{1\leq j<k}|Y\prime_{0}=1)$$

which are approximated based on ${\left\{\gamma_{k}\right\}}_{1\leq k<M}$, ${\left\{\gamma_{3\prime ,k}\right\}}_{0\leq k<M}$ and ${\left\{\gamma_{5\prime ,k}\right\}}_{1\leq k<M}$ (see Supplementary Notes).

For convenience, we compute the probability of an overlapping hit (across all possible overlap positions) as

(18) $$\beta :=\sum_{j=1}^{M-1}\beta_{j},\;\beta_{3\prime }:=\sum_{j=0}^{M-1}\beta_{3\prime ,j},\;\beta_{5\prime }:=\sum_{j=1}^{M-1}\beta_{5\prime ,j}$$

which makes use of the fact that (14)–(17) represent mutually exclusive events.

### 2.4 Distribution of the clump size

Next, we describe a recursive approach of computing the clump size distribution, ${\left\{\theta_{c}\right\}}_{c>0}$, which is similar to the approach described in Pape *et al.* (2008). The main difference relative to the original approach is that it utilizes Definitions (18), instead of the *marginal overlapping hit probabilities*.

#### *2.4.1* Clump size probability when scanning a single strand

We shall first derive the clump size probability for the simple case of scanning a single strand for TFBSs and discuss an extension to scanning both strands in the next section.

A clump of size one is defined as a single motif hit that does not overlap any other motif hits (before or after the clump start) (Pape *et al.*, 2008). Its probability is given by

(19) $$\theta_{1}:=P({\left\{Y_{i}=0\right\}}_{1\leq i<M}|Y_{0}=1,{\left\{Y_{-j}=0\right\}}_{1\leq j<M})$$

where we assume a hit $Y_{0}=1$ and no further overlapping hits upstream $Y_{-j}$ and downstream *Y_j_*.

Unfortunately, the exact computation of Definition (19) is intractable. However, it is possible to approximate this quantity. To this end, without loss of generality we order the motif hits that occur in a clump from left to right such that the first hit is never overlapped by any upstream hit. Then, assuming that we start from the first hit, the probability of observing no further downstream overlapping hits equals

(20) $${\tilde{\theta }}_{1}=P({\left\{Y_{i}=0\right\}}_{1\leq i<M}|Y_{0}=1)=1-\beta$$

where we used Definition (18).

Subsequently, we recursively define the proportion of obtaining a clump of size *c* > 1 by dividing out the original end of the *c* – 1-clump, extending an overlapping hit *downstream* of the last hit and multiplying in the new end of the clump, which yields

(21) $${\tilde{\theta }}_{c}=\frac{{\tilde{\theta }}_{c-1}(1-\beta )\beta }{1-\beta }={\tilde{\theta }}_{c-1}\times \beta .$$

Consequently, note that the clump size is geometrically distributed.

Finally, the clump size probabilities are obtained according to

(22) $$\theta_{c}=\frac{{\tilde{\theta }}_{c}}{\sum_{j>0}{\tilde{\theta }}_{j}}$$

#### *2.4.2* Clump size probability when scanning both strands

Next, we derive the clump size distribution when both DNA strands are scanned for motif hits.

As above, we start by defining the 1-clump probability, which might exhibit a respective forward or reverse strand hit with probability

(23) $$\begin{array}{cc} \theta_{1}^{f} & :=P({\left\{Y_{i}=0,Y\prime_{i}=0\right\}}_{1\leq i<M},Y\prime_{0}=0|\\ & \;\;Y_{0}=1,{\left\{Y_{-i}=0,Y\prime_{-i}=0\right\}}_{1\leq i<M}) \end{array}\;$$

(24) $$\begin{array}{cc} \theta_{1}^{r} & :=P({\left\{Y_{i}=0,Y\prime_{i}=0\right\}}_{1\leq i<M}|\\ & \;\;Y\prime_{0}=1,Y_{0}=0,{\left\{Y_{-i}=0,Y\prime_{-i}=0\right\}}_{1\leq i<M}) \end{array}$$

Without loss of generality, we order the sequence of events according to $Y_{1}Y\prime_{1}Y_{2}Y\prime_{2}\cdots$ and count the hits in a clump in that order from left to right. Assuming that we encounter the first hit in the clump, the probability that it is not overlapped by any downstream (or palindromic) hits is given by

(25) $${\tilde{\theta }}_{1}^{f}:=1-\beta -\beta_{3\prime }\;\;\text{and}\;\;{\tilde{\theta }}_{1}^{r}:=1-\beta -\beta_{5\prime }.$$

where we made use of Definitions (18).

We proceed by recursively defining the proportion of obtaining a clump of size *c* > 1 by dividing out the original end of the *c* – 1-clump, extending an overlapping hit *downstream* of the last hit and multiplying in the new end of the clump, which leads to the following formula

(26) $$\left[\begin{array}{c} {\tilde{\theta }}_{c}^{f}\\ {\tilde{\theta }}_{c}^{r} \end{array}\right]=\left[\begin{array}{ccc} \beta & & \frac{{\tilde{\theta }}_{1}}{\tilde{\theta }\prime_{1}}\beta_{5\prime }\\ \frac{\tilde{\theta }\prime_{1}}{{\tilde{\theta }}_{1}}\beta_{3\prime } & & \beta \end{array}\right]\cdot \left[\begin{array}{c} {\tilde{\theta }}_{c-1}^{f}\\ {\tilde{\theta }}_{c-1}^{r} \end{array}\right].$$

Finally, we obtain the clump size probabilities *θ_c_* regardless of the strandedness of the last hit by

(27) $$\theta_{c}=\frac{{\tilde{\theta }}_{c}^{f}+{\tilde{\theta }}_{c}^{r}}{\sum_{i>0}{\tilde{\theta }}_{i}^{f}+{\tilde{\theta }}_{i}^{r}}.$$

### 2.5 Comparison between methods

We estimated background models of various orders from a subset of Dnase-I hypersensitive sites published by the ENCODE consortium (Thurman *et al.*, 2012) as such sequences are frequently under scrutiny when it comes to searching for motif matches.

**Fig. 2.**
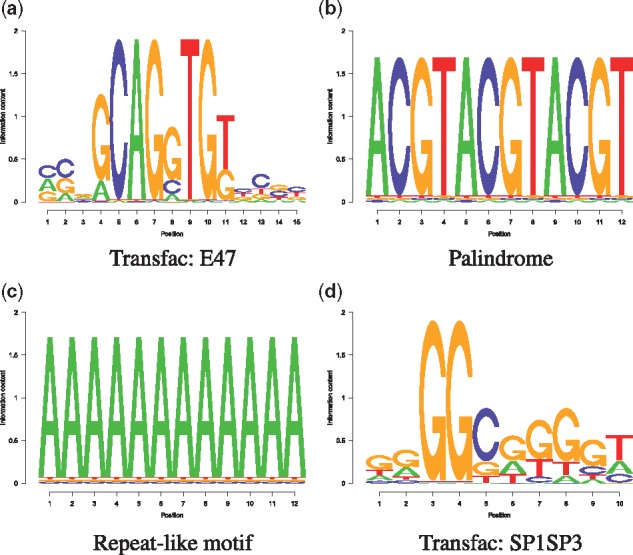
DNA motifs

We compared the models for (i) different sequence lengths, (ii) different false positive probabilities *α* of obtaining a motif hit, (iii) different background model orders *d* and (iv) various motifs (see Fig. 2(a–c)). A summary of the setup is given in Table 1.

**Table 1.** Comparative analysis

d	α	seqlen
0	0.01	1 kb
0	0.001	10 kb
1	0.001	10 kb
2	0.001	10 kb

*Note*: Analysis setup which is used for all motifs.

As a reference for the analysis, we determined an empirical distribution *P_E_* by sampling 100 000 random DNA sequences from the background models and counted the number of respective motif hits, which resulted in a highly reproducible empirical distribution. In order to visualize the sampling noise, we additionally split the 100 000 samples into 100 batches consisting of 1000 sequences each and determined the 25 and 75% percentiles for each *x* of $P_{E}(X=x)$ over the batches.

For the comparison, we invoked the new compound Poisson approximation $P_{CP}^{N}$ (as described above), the previous compound Poisson model $P_{CP}^{P}$ (Pape *et al.*, 2008) and the binomial model *P_Bin_*, which is defined by

$$P_{Bin}(X=x)=\left(\begin{array}{c} 2\times (N-M+1)\\ x \end{array}\right)\alpha^{x}{\left(1-\alpha \right)}^{2\times (N-M+1)-x}.$$

In order to allow for a fair comparison, we slightly changed the original compound Poisson model $P_{CP}^{P}$ (see Supplementary Notes).

The performances of $P_{CP}^{N}$, $P_{CP}^{P}$ and *P_Bin_*, were measured by the total variation distance relative to *P_E_* using

(28) $$d(P_{E},Q)=\sum_{x\geq 0}|P_{E}(x)-Q(x)|$$

where *Q* denotes a placeholder for the approximative models. Additionally, we measure the discrepancy on the 5% significance region only

(29) $$d_{5\%}(P_{E},Q)=\sum_{x\geq q_{95\%}}|P_{E}(x)-Q(x)|.$$

where $q_{95\%}$ denotes the 95%-percentile of *P_E_*.

Finally, we compared the previous clump size approximation (Pape *et al.*, 2008) and the novel approximation (see Section 2.4.2) by measuring their total variation distances to an empirical clump size distribution, which was generated by counting clump size occurrences in a random 10 Mb sequence drawn from the background.

### 2.6 Influence of higher-order background models on motif enrichment

We downloaded human CpG islands from the UCSC genome browser (Kent *et al.*, 2002) and estimated background models of order d∈{0,1,2}. The SP1SP3 motif was obtained from Transfac (Wingender *et al.*, 1996) (see Fig. 2d).

We studied the distribution of the number of motif hits for sequences of length 10 kb with α=0.01 for different background orders *d*. The compound Poisson approximation with fixed order *d*, denoted $P_{CP,d}^{N}$, was determined as described above, where *d* explicitly indicates which background order was used. We determined two different variants of the sampling-based distribution: First, we computed a sampling-based distribution where the sequences were generated according to the background orders d∈{0,1,2}, but where the score is always evaluated w.r.t. order *d* = 0, denoted by $P_{E,d,0}$. This renders $P_{E,d,0}$ comparable to $P_{CP,0}^{N}$ and allows us to measure the discrepancy between the distributions for different *d*. Second, we determined a sampling-based distribution with matched background orders *d* for the sequence generating process and the score computation, denoted by $P_{E,d,d}$.

### 2.7 Comparison of the models on JASPAR motifs

We compared $P_{CP}^{N},\;P_{CP}^{P}$ and *P_Bin_* on all JASPAR 2014 motifs with a minimum length of 6 bps (578 motifs in total). To this end, we estimated an order-1 background model on a subset of ENCODE Dnase-I hypersensitive sites (as described above). We analyzed the number of motif hits in sequences of length 10 kb with α=0.001. As a reference, we determined the sampling-based distribution *P_E_*. To assess how $P_{CP}^{N}$ compares to the other models we determined

(30) $$\Delta d_{N-P}=d(P_{CP}^{N},P_{E})-d(P_{CP}^{P},P_{E})$$

(31) $$\Delta d_{N-B}=d(P_{CP}^{N},P_{E})-d(P_{Bin},P_{E})$$

for each motif.

## 3 Results

### 3.1 Comparison between various motif count models

In this Section, we assess the adequacy of the analytic models $P_{CP}^{N},\;P_{CP}^{P}$ and *P_Bin_* with respect to their discrepancy to *P_E_*. To this end, we study a range of motif structures (see Fig. 2a and b) and parameter settings (see Table 1).

**Fig. 3.**
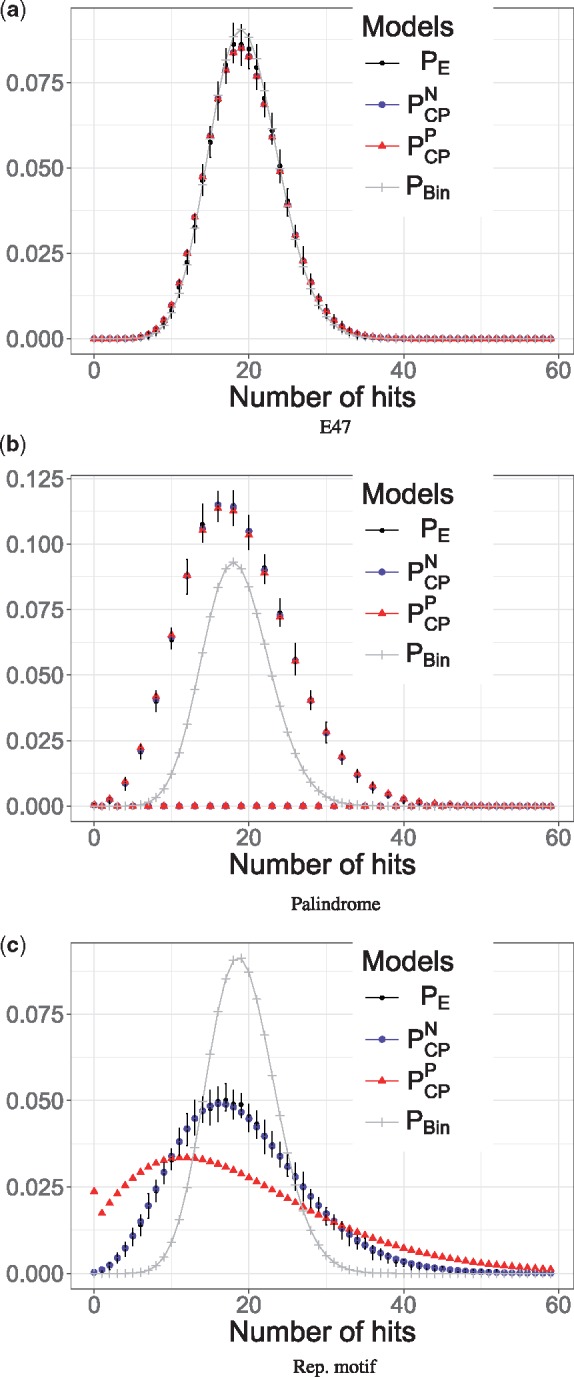
Comparison between the methods for different motif types: Each panel shows the distribution of the number of motif hits in 10 kb sequences generated from an order-1 background model using $\alpha =10^{-3}$ for the motifs depicted in Figure 2a, b and c, respectively. For all cases, the empirical (black), the new (blue) and the previous compound Poisson approximation (red) and the binomial approximation (gray) are depicted. Moreover, the empirical distribution was augmented by error bars showing the 25–75% quantiles to depict the sampling noise relative to batches consisting of 1000 sequences

The shape of the distribution of the number of motif hits depends on the structure of the motif. Accordingly, self-overlapping motifs (such as palindromes and repeat-like motifs) generally lead to an increased variance compared to non-self-overlapping motifs (compare *P_E_* in Fig. 3b and c) against (a)). Moreover, for palindromic motifs in particular, the number of hits must be a multiple of two, as a motif hit is always paired with a hit on the reverse strand (see Fig. 3b).

As described previously (Rahmann *et al.*, 2003), provided that motif hits occur only rarely, the binomial model establishes an accurate approximation of the *P_E_* for non-self-overlapping motifs (see Fig. 3a). However, due to its inherent independence assumption it is not suitable for self-overlapping motifs (Pape *et al.*, 2008). *P_Bin_* systematically underestimates the variance compared to *P_E_* which would lead to an excess of false positives for the enrichment test (compare *P_Bin_* with *P_E_* in Fig. 3b and c).

By contrast, $P_{CP}^{N}$ and $P_{CP}^{P}$ take the self-overlapping structures explicitly into account, which in principle makes them suitable for all motif structures. Therefore, they respond with an increased variance for self-overlapping motifs (see Fig. 3b and c), while for non-self-overlapping motifs they lead to a comparably narrow distribution (see Fig. 3a). While, for non-self-overlapping motifs, all approximations perform similarly (see Fig. 3a), in particular, for palindromes, only $P_{CP}^{N}$ and $P_{CP}^{P}$ achieve accurate approximations, as they render odd numbers of hits impossible (see Fig. 3b). On the other hand, we find a discrepancy between $P_{CP}^{N}$ and $P_{CP}^{P}$ for the repeat-like motif. $P_{CP}^{P}$ overestimates the variance compared to *P_E_*, while $P_{CP}^{N}$ matches *P_E_* vary closely (see Fig. 3c). This difference can be attributed to the refined estimation of the clump size probabilities *θ_c_* via the *principal overlapping hit probabilities* as opposed to the inherently redundant *marginal overlapping hit probabilities*.

Next, we assess the accuracies of the models across all parameter settings (see Table 1) using Equation (28). In general, we find largely concordant results across the parameters (see Tables 2–4). That is, in most cases, $P_{CP}^{N}$ achieves equally accurate or better solutions compared to $P_{CP}^{P}$ and *P_Bin_*. A notable exception to this rule represents the non-self-overlapping motif for a relaxed significance level α=0.01. In that situation, *P_Bin_* compares favorably to $P_{CP}^{N}$ and $P_{CP}^{P}$, because the compound Poisson models overestimate the variance relative to the reference (see row 1 in Table 2). The reason for this is the violation of the ‘rare hit’ assumption (Reinert *et al.*, 2000). While, this assumption applies to the binomial and the compound Poisson model, the compound Poisson model responds more sensitively to its violation. Hence, the discrepancy. Prescribing a stringent *α* (e.g. α=0.001) largely eliminates this effect in which case $P_{CP}^{N},\;P_{CP}^{P}$ and *P_Bin_* yield comparable results (see row 2–4 in Table 2).

**Table 2.** Performance comparison for E47 (see Fig. 2a)

D	α	Seqlen	$d(P_{E},P_{CP}^{N})$	$d(P_{E},P_{CP}^{P})$	$d(P_{E},P_{Bin})$
1	10^–2^	1000	0.211	0.233	0.0864
0	10^–3^	10 000	0.0285	0.0291	0.0401
1	10^–3^	10 000	0.032	0.0325	0.0386
2	10^–3^	10 000	0.0289	0.0293	0.0411

**Table 3.** Performance comparison for the palindrome (see Fig. 2b)

D	α	Seqlen	$d(P_{E},P_{CP}^{N})$	$d(P_{E},P_{CP}^{P})$	$d(P_{E},P_{Bin})$
1	10^–2^	1000	0.0948	0.119	1
0	10^–3^	10 000	0.00923	0.016	1
1	10^–3^	10000	0.0108	0.0214	1
2	10^–3^	10 000	0.0143	0.0235	1

**Table 4.** Performance comparison for the repeat-like motif (see Fig. 2c)

d	α	Seqlen	$d(P_{E},P_{CP}^{N})$	$d(P_{E},P_{CP}^{P})$	$d(P_{E},P_{Bin})$
1	10^–2^	1000	0.0656	0.824	0.735
0	10^–3^	10 000	0.0177	0.467	0.605
1	10^–3^	10 000	0.0191	0.464	0.611
2	10^–3^	10 000	0.0194	0.5	0.63

Next, we inspect the performance with respect to different background model orders. We find that the relative accuracies between the models largely remain preserved across different orders *d* (see rows 2–4 in Tables 2–4). This underlines the adequacy of employing general higher-order background models in our setting.

As the right tail of the distribution influences the motif hit enrichment test the most, we investigated the accuracy of the approximation specifically in the tail. Since, the empirical distribution cannot be used to assess extremely rare events, we chose to assess the accuracies of the models on the 5% significance region using Equation (29), for which *P_E_* can be estimated highly reproducibly. We observe that the relative performances are in high agreement with the assessment of the entire distribution using Equation (28) (see Supplementary Tables S1–S3 and Tables 2–4). In other words, we do not find cases, where the discrepancy measured with Equation (28) and (29) disagree substantially.

Finally, we investigated the accuracy of the clump size approximations *θ_c_* that arise from the previous (Pape *et al.*, 2008) and our new method (see Section 2.4.2). While, the previous model achieves a slightly more accurate clump size approximation relative to the new model for the non-self-overlapping and the palindromic motif, the absolute differences are nevertheless comparable (see Supplementary Figs S6a–S6b and Table S4). This is in agreement with the similar results observed for the respective compound Poisson models (see Fig. 3a and b). On the other hand, for the repeat-like motif, the previous clump size model clearly overestimates the width of the empirical clump size distribution, whereas the new model seems to capture its shape more accurately (see Supplementary Fig. S6c and Table S4).

### 3.2 Influence of higher order background models

In this section, we address the question of how an inappropriate background model choice might influence the distribution of the number of motif hits and thus the statistical enrichment test. To this end, we count *SP1SP3* motif occurrences (see Fig. 2d) in human CpG regions.

We first emulate the effect of assuming an order-0 background model while the actual sequence generating process is driven by a potentially more complex order-*d* Markov model with d∈{0,1,2,3}. This scenario simulates the effect of employing a too simplistic model to recapitulate (perhaps more complicated) real-world observations (e.g. real promoter sequences).

**Fig. 4.**
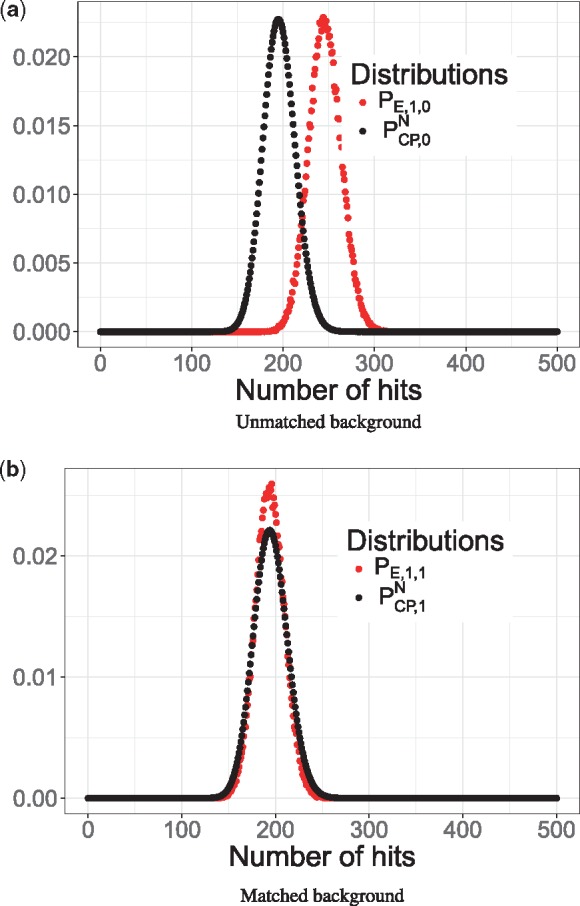
Motif hit count distribution under the influence of assuming an order-0 and order-1 background model to investigate CpG islands. (**a**) The compound Poisson approximation (black) assumes an order-0 background, while the empirical distribution (red) was generated by an order-1 background model, This introduces a discrepancy between the distributions explained by commonly occurring dinucleotide which are ignored by the order-0 background. (**b**) The compound Poisson approximation (black) accounts for the order-1 background, which leads to a more accurate compound Poisson approximation relative to the empirical distribution

If the sequence is generated by a simple order-0 model, that is the model assumption matches the ‘true’ sequence generating process, as expected, $P_{E,0,0}$ and $P_{CP,0}^{N}$ are in high agreement (see Supplementary Fig. S2a). However, if the sequence is generated by a higher-order background model with *d* > 0 (against the assumption of observing an order-0 complexity sequence), $P_{E,d,0}$ and $P_{CP,0}^{N}$ become increasingly discordant (see Fig. 4a and Supplementary Fig. S1). $P_{CP,0}^{N}$ underestimates the number of motif hits compared to $P_{E,d,0}$, because it ignores higher-order sequence features, and in particular, commonly occurring ’C’ and ’G’ repeats, which are also characteristic for the SP1SP3 motif. We notice that the discrepancy is dominated by ignoring dinucleotide frequencies (see Fig. 4a), which induces a substantial shift between $P_{CP,0}^{N}$ and $P_{E,1,0}$. Adopting even higher-order models (*d* = 2 and *d* = 3) lead to only a slight further increase in the shift (see Supplementary Fig. S1a and S1b). Therefore, if $P_{CP,0}^{N}$ is used as the basis for an enrichment test, an excess of false positive predictions would be incurred as the number of hits is substantially underestimated by the order-0 background.

In contrast, properly accounting for higher-order sequence features, by generating the sequence for $P_{E,d,d}$ and $P_{CP,d}^{N}$ with matched background orders eliminates the discrepancy between the distributions even for orders *d* > 0 (see Fig. 4b and Supplementary Fig. S2 for *d* = 1 and *d* > 1, respectively).

### 3.3 Differences in performance for Jaspar motifs

In this section, we compare $P_{CP}^{N},\;P_{CP}^{P}$ and *P_Bin_* on all 578 known Jaspar 2014 motifs (Sandelin *et al.*, 2004). First, we measured the total variation for all motifs according to (28) between approximative models and *P_E_*. As expected, for the majority of the motifs, the models reach similar conclusions and therefore similar discrepancy measures (see Supplementary Fig. S3). Next, to quantify the relative accuracies of $P_{CP}^{P}$ and *P_Bin_* compared to $P_{CP}^{N}$ we measured the differences of the total variances according to Equation (30) and (31). Using the Wilcoxon rank sum test, we found that across all Jaspar motifs, $P_{CP}^{N}$ significantly improves over *P_Bin_* (*P-value*<2.2e−16) as well as over $P_{CP}^{P}$ (*P-value*=0.00028).

Motifs for which the models disagree the most correspond to self-overlapping motifs. Examples of which include palindromes, like *PDR3*, *MYC3*, *PHO4*, *gt* and *LFY*, when comparing $P_{CP}^{N}$ and *P_Bin_* (see Supplementary Fig. S4), and repeat-like motifs, like *DAF-12*, *EWSR1-FLI1*, *NHP6A & B*, *SFP1* and *SOC1* for $P_{CP}^{N}$ and $P_{CP}^{P}$ (see Supplementary Fig. S5).

## 4 Discussion

We presented an improved compound Poisson model based on Pape *et al.* (2008). This model facilitates motif hit enrichment testing, by comparing the observed number of motif hits in a given DNA sequence to the numbers that would emerge in sequences that are produced by a background model. As in the original model, the improved model also considers binding site predictions on both DNA strands. Furthermore, we proposed two major improvements over the original model: First, we considered general order-*d* background models, as opposed to an order-0 background, to capture the properties of unbound sequences. While, order-0 background models (Grant *et al.*, 2011; Pape *et al.*, 2008; Rahmann *et al.*, 2003; Roider *et al.*, 2007), have been widely used due to their inherent simplicity, they ignore higher-order sequence features (e.g. CpG frequencies) and may therefore be inappropriate for studying naturally occurring DNA sequences. General order-*d* background models are capable of capturing, e.g. dinucleotide frequencies, which is important to describe CpG islands, that frequently overlap with regulatory regions.

Second, we developed a novel approach for approximating the so-called *principal overlapping hit probabilities*. We argue that those give more accurate results for estimating the clump size distribution. By contrast, the *marginal overlapping hit probabilities*, which were used earlier (Pape *et al.*, 2008), describe overlapping hits redundantly, and are therefore prone to overestimate the clump size distribution (especially for repeat-like motifs).

We systematically compared the compound Poisson models and the binomial model for a range of parameter settings and motif types. Our results suggest that the improved compound Poisson approximation generally yields similar or more accurate approximations compared to the other models, provided that motif hits occur only rarely (Reinert *et al.*, 2000). We have demonstrated that when scanning for motif matches with $\alpha =10^{-3}$ (or more stringent *α*), the ‘rare hit’ assumption is largely met, whereas for a relaxed significance level of $\alpha =10^{2}$, the compound Poisson approximation tends to mildly overestimate the variance. However, we suggest that for $\alpha =10^{-2}$ the approximation may be still useful, since it results in a slightly broader (conservative) approximation rather than a distribution that is too narrow. For larger *α* (e.g. α=0.1), motif matches would occur too frequently for the compound Poisson approximation to be reasonably applicable. However, such large choices for *α* are not supported by the frequencies at which TFBSs are found in the genome, anyway, because TFs tend to bind to a comparably small proportion of the genome.

We demonstrated the relevance of using higher-order background models for enrichment testing by counting *SP1SP3* binding sites in human CpG islands, since CpGs are frequently found in regulatory regions (e.g. promoters). Ignoring higher-order sequence features in the background model might incur biases that can lead to an excess of false positive predictions and bears the caveat of reaching false conclusions. On the other hand, such biases may be substantially reduced by utilizing a general order-*d* background model.

The choice of background model and order is, however, in itself a difficult question, hard to answer in general. In principle, it is conservative to model the group of sequences under study as background. That is, if one searches for motifs in promoters, one should compare to a background that mimics promoters rather than coding sequence. The latter choice would inflate the significance of the promoter motifs. Selecting an appropriate Markov model order has been dealt with e.g. based on a Chi-square test for independence (Reinert *et al.*, 2000) or using the BIC criterion (Csiszár *et al.*, 2000). For the purposes of estimating statistical significance we think it is reasonable not to emulate the sequences in too much detail (by choosing a high order *d*) since the searched motif would be captured by the background, which would in turn make it appear to be insignificant. Thus, we recommend to choose an order of maximally *d* = 2 because this captures well the known biological effects, namely CpG islands.

Lastly, we showed that across a large set of known motifs (from Jaspar 2014 (Sandelin *et al.*, 2004)), the new compound Poisson approximation yields similar or better accuracies compared to the other models, which underlines the relevance of our approach.

## Supplementary Material

Supplementary Data
