# Supplementary material for: An improved compound Poisson model for the number of motif hits in DNA sequences
Source: Bioinformatics. 2017 Aug 28;33(24):3929–37. doi: 10.1093/bioinformatics/btx539 (PMC5860096; doi:10.1093/bioinformatics/btx539)
Supplement: Supplementary Data [file btx539_supplementary_new_figurenumbering.pdf]

Supplementary Notes

1 Estimating the background model

We obtain the transition probabilities of the order-d background model by a maximum likelihood procedure (Reinert *et al.*, 2000) under the constraint that words on both strands as well as with reversed nucleotide orders (from 5' to 3' and 3' to 5') to occur equally likely. To that end, we make use of the detailed balance equations

$$\mu(a_0 \cdots a_{d-1})\pi(a_0 \cdots a_{d-1}; a_d) = \mu(a'_0 \cdots a'_{d-1})\pi(a'_0 \cdots a'_{d-1}; a'_d) \tag{1}$$

$$\mu(a_d \cdots a_1)\pi(a_d \cdots a_1; a_0) = \mu(a_0 \cdots a_{d-1})\pi(a_0 \cdots a_{d-1}; a_d) \tag{2}$$

where a'_i denotes the complementary nucleotide of a_i. As a consequence of requiring Equation (1)-(2) to hold we obtain the following counting function

$$\begin{aligned} N(a_0 \cdots a_{d-1}, a_d) = \sum_{i=1}^{N-d} & \mathbf{1}[w_i \cdots w_{i+d} = a_0 \cdots a_d] + \\ & \mathbf{1}[w'_i \cdots w'_{i+d} = a_0 \cdots a_d] + \\ & \mathbf{1}[w_{i+d} \cdots w_i = a_0 \cdots a_d] + \\ & \mathbf{1}[w'_{i+d} \cdots w'_i = a_0 \cdots a_d]. \end{aligned} \tag{3}$$

where $\mathbf{1}[]$ denotes the indicator function which evaluates to one if its argument is true. $N(a_0 \cdots a_{d-1}, a_d)$ is then used to estimate the transition probabilities (see Equation (1) in the main text).

From the acquired estimates of the transition probabilities $\hat{\pi}$, we obtain the stationary probabilities $\mu(a_1 \cdots a_d)$ by utilizing the power method for eigenvalue decomposition (Karlin and Taylor, 1981).

2 The score distribution

In this section, we discuss a recursive algorithm for computing the distribution of the scores $P(S = s)$. Originally an algorithm that assumes an underlying order-0 background model was described by several groups (Rahmann *et al.*, 2003; Grant *et al.*, 2011; Touzet *et al.*, 2007). It was also pointed out that this algorithm can be extended to general order-d background models (Beckstette *et al.*, 2006; Touzet *et al.*, 2007). An implementation of such an algorithm was reported in Thomas-Chollier *et al.*, 2008, without describing the full details of the implementation. Therefore, we state the algorithm for computing the score distribution based on an order-d background model in this section.

2.1 Discretization of the score range

In order to derive an efficient algorithm for the score distribution $P(S = s)$ it is necessary to discretize the real-valued score range. As a consequence, the score distribution will not be exact, with its accuracy depending on discretization granularity which has to be chosen as a compromise between accuracy and runtime efficiency.

To discretize the score range, we determine the maximally and minimally achievable score, denoted by s_{max} and s_{min}, for the given PFM of length M and a background model with order d. s_{max} and s_{min} can be obtained efficiently in $O(M|\mathcal{A}|^{d+1})$. Subsequently, the score range is split into G equally spaced intervals using a predefined score granularity Δs according to

$$G = \frac{s_{max} - s_{min}}{\Delta s}.$$

Our decision to use the natural logarithm to obtain the motif score in Equation (2) (see main text) influences the choice of the score granularity Δs. Note, however, that since the logarithm is a monotonic function, one can find a corresponding score granularity also for a different logarithm base (e.g. $base = 2$) which would yield a similar discretization error. Hence, the choice of the logarithm base does not affect the results.

For all experiments discussed in the main article, we used a score granularity of $\Delta s = 0.1$ which seems to be a reasonable compromise between accuracy and runtime efficiency.

2.2 Computation of the score distribution

Similarly as described in (Rahmann *et al.*, 2003), we seek to establish the distribution of the scores by a recursive procedure. To this end, we successively determine the score distribution Q_j of a sub-motif ranging from position 1 through j by reusing the score distribution up to the previous position Q_{j-1} and incorporating the scores at position j. In addition to that the distribution must be computed with respect to all possible prefix words of length d in order to correctly account for local dependencies between neighboring nucleotides in an order-d background model.

We define the initial score distribution at position $c = \max(1, d)$ for all prefix words $a_1 \cdots a_c \in \mathcal{A}^c$ and all discrete scores s ranging from s_{min} to s_{max} using the stationary distribution μ of the background model according to

$$Q_c(s, a_1 \cdots a_c) := \mathbf{1}[l_d(a_1 \cdots a_c) = s]\mu(a_1 \cdots a_c)$$

where we made use of the discretized score l_c w.r.t. the first c positions of the motif defined by

$$l_c(a_1 \cdots a_c) := \left\lfloor \log \left(\frac{\prod_{i=1}^{c} p_i(a_i)}{\mu(a_1 \cdots a_c)} \right) \right\rfloor .$$

The floor operator denotes the discretization of the scores.

Next, we define the score distribution Q_j recursively by extending Q_{j-1} the score contributions at position j. Therefore, we evaluate the following equation for all words $a_1 \cdots a_d a_{d+1} \in \mathcal{A}^{d+1}$ and for all discrete scores s ranging from s_{min} to s_{max} using the transition probabilities of the background model π according to

$$Q_j(s, a_2 \cdots a_{d+1}) := \sum_{a_1 \in \mathcal{A}} Q_{j-1}(s - l_j(a_1 \cdots a_d, a_{d+1}), a_1 \cdots a_d) \times \pi(a_1 \cdots a_d; a_{d+1}). \tag{4}$$

Here we made use of the discretized score contribution at position j defined by

$$l_j(a_1 \cdots a_d, a_{d+1}) := \left\lfloor \log \left(\frac{p_j(a_{d+1})}{\pi(a_1 \cdots a_d; a_{d+1})} \right) \right\rfloor . \tag{5}$$

The score distribution is determined in an ordered fashion starting with $j = \max(1, d)$ and terminates when $j = M$. Finally, we obtain the score distribution by averaging over all prefix words $a_1 \cdots a_d \in \mathcal{A}^d$

$$P(s) = \sum_{a_1 \cdots a_d} Q_M(s, a_1 \cdots a_d).$$

2.3 Runtime

The runtime of the algorithm for computing the score distribution is given by $O(M|\mathcal{A}|^{d+1}G)$, where M denotes the length of the motif, $|\mathcal{A}|$ the alphabet size, d the background order and G the number of bins for discretizing the score range.

3 The joint distribution of a pair of scores

In this section, we describe the computation of the joint distribution of the scores $P(S_0 = s, S_k = s')$ for two motif start positions 0 and k. The algorithm assumes that the underlying DNA sequence was generated by a order-d Markov model. This algorithm extends the originally proposed algorithm (Pape *et al.*, 2008) which built on the assumption of an order-0 background. On the other hand, the algorithm for computing $P(S_0 = s, S_k = s')$ accounts for the prefix words of length d in a similar manner as the algorithm for computing $P(S = s)$ (see above).

We shall ultimately use the algorithm to acquire the marginal overlapping hit probabilities that were described in the main text.

3.1 Computation of the joint score distribution

Along the line of the previous section, it is necessary to discretize the score range with a predefined score granularity Δs. For this purpose, we use the same granularity to discretize the scores s and s' which always was chosen to be $\Delta s = 0.1$ for the experiments in this article.

Next, we define the *local score contributions* in as similar way as in the previous section as

$$l_j(a_1, \cdots, a_d, a_{d+1}) := \begin{cases} \left\lfloor \log\left(\frac{\prod_{i=1}^d p_i(a_i)}{\mu(a_1 \cdots a_d)}\right) \right\rfloor & \text{if } j = d \\ \left\lfloor \log\left(\frac{p_j(a_{d+1})}{\pi(a_1 \cdots a_d; a_{d+1})}\right) \right\rfloor & \text{if } d < j \leq M \\ 0 & \text{otherwise.} \end{cases}$$

In order to keep the notation uncluttered, we shall use l_j in the following without explicitly indicating its dependence on the sequence $a_1 \cdots a_{d+1}$.

We proceed by deriving an recursive algorithm, similarly as described in the previous section.

We initialize the score distribution at position $c = \max(1, d)$ for all prefix words $a_1 \cdots a_c \in \mathcal{A}^c$ and for all discrete scores s and s' that range from the respective minimally and maximally achievable scores using

$$Q_c(s, s', a_1 \cdots a_c) := \sum_{a_1 \in \mathcal{A}} \mathbf{1}[s = l_c]\mathbf{1}[s' = l_{c-k}] \times \mu(a_1 \cdots a_c). \tag{6}$$

Subsequently, Q_j is recursively expressed using Q_{j-1} and the score contribution at position j. Therefore, for all words $a_1 \cdots a_{d+1} \in \mathcal{A}^{d+1}$ and for all discrete scores s and s' ranging from the respective minimally and maximally achievable scores, we evaluate

$$Q_j(s, s', a_2 \cdots a_{d+1}) = \sum_{a_1 \in \mathcal{A}} Q_{j-1}(s - l_j, s' - l_{j-k}, a_1 \cdots a_{d+1}) \times \pi(a_1 \cdots a_d; a_{d+1}) \tag{7}$$

where π denotes the transition probability of the order-d background model.

We evaluate the score distribution in an ordered fashion by starting $Q_{\max(1,d)}$ and iterating the process until we obtain Q_{M+k}. Finally, we obtain the joint score distribution by averaging over all prefix words $a_1 \cdots a_d$

$$P(S_0 = s, S_k = s') = \sum_{a_1 \cdots a_d \in \mathcal{A}^d} Q_{M+k}(s, s', a_1 \cdots a_d)$$

3.2 Scanning both strands

As we are interested in finding motif hits on both strands of the DNA, we scan the sequence once with the original motif and once with the reverse complemented motif. Therefore, analogously to the local score contributions for the forward strand l_j, we introduce the local score contributions on the reverse strand as l'_j. l'_j is obtained by considering the reverse complemented instead of the original motif. Otherwise, the algorithm remains the same.

3.3 Runtime

The runtime of the algorithm presented above is $O(M|\mathcal{A}|^{d+1}G^2)$, where M denotes the motif lengths, $|\mathcal{A}|$ the alphabet size, and G the granularity of the score range.

However, there are a number of ways how the algorithm can be optimized: Firstly, and most importantly, after each iteration i with $1 \leq i \leq M + k$ any probability mass associated with scores that are certainly below t_α after the final iteration can

be dropped. Similarly, any probability mass that is certainly above t_α after the final iteration can be aggregated (Pape *et al.*, 2008). Both of these considerations reduce the region over which to perform the convolution and thereby the number of operations substantially. Secondly, we keep the motif sub-score distributions for each position when computing the score distribution at one position and reuse them for computing the joint distribution of scores. A similar approach was also proposed by Pape *et al.*, 2008. This avoids redundant computational effort for initializing the 2D arrays. Third, we propose a novel optimization strategy: We initialize the joint distribution of scores by means of an enumerative approach (that iterates over all words) for the initial $m \leq M + k$ positions in the motif. While, the asymptotic runtime of the enumerative approach depends exponentially on m, it might still be efficient to use for small m, because for the first few positions, most of the entries in $Q_i(s, s', w_i \cdots w_{i+d-1})$ are zero. In that case, computational resources are wasted by multiplications with zeros, while the enumerative method more efficiently populates $Q_i(s, s', w_i \cdots w_{i+d-1})$ with non-zero entries. We compute optimal subsequence length m, up to which point the enumerative approach can be done faster than the recursive approach by summing up the number of multiplications that are necessary for each approach.

4 Principal overlapping hit probabilities

In this section, we derive an approximation of the *principal overlapping hit probabilities* that were introduced in the main paper.

The main difference of the *principal overlapping hit probabilities* compared to the *marginal overlapping hit probabilities* is that the former ones explicitly exclude intermediate motif hits. Therefore, the *principal overlapping hit probabilities* quantify the probability of obtaining a motif hit non-redundantly, whereas the *marginal overlapping hit probabilities* might contain redundant information about overlapping motif hits.

4.1 Overlapping hits with respect to a single DNA strands

For simplicity, we first derive a recursive approach for computing the overlapping hit probability with respect to scanning only a single strand. First, we introduce the

base case

$$\beta_1 := \gamma_1 = P(Y_1 = 1 | Y_0 = 1) \tag{8}$$

for which there exists no intermediate event.

Subsequently, we derive a recursive approximation for β_k for $k > 1$. The approximation rests on the assumption that having observed $Y_m = 1$, the events Y_l and Y_n are statistically independent of one another for $l < m < n$. That is, events prior to and after a motif hit are independent. Note that this assumption is exactly met if the TFBSs can be described by a single word. If multiple words are compatible with the TF motif under the given score threshold, this assumption holds true only approximately.

Theorem 1. *Assuming that the events Y_l and Y_n are independent if $Y_m = 1$, the principal overlapping hit probability can be expressed as*

$$\beta_k = \gamma_k - \sum_{i<k} \beta_i \gamma_{k-i} \tag{9}$$

where $l < m < n$ and $k > 1$.

Proof. We shall proof Equation (9) by induction. The base case β_1 is given by Equation (8). Next, assuming that we have already obtained $\{\beta_i\}_{1 \le i < k}$, we show that the following expression holds true

$$\gamma_k = \beta_k + \sum_{i<k} \beta_i \gamma_{k-i}.$$

We proceed as follows

$$\gamma_k = P(Y_k = 1 | Y_0 = 1)$$

By Definition 1 in the main text

$$= \sum_{y_1 \cdots y_{k-1} \in \{0,1\}^{k-1}} P(Y_k = 1, y_1 \cdots y_{k-1} | Y_0 = 1)$$

to indicate that $Y_1 \cdots Y_{k-1}$ where averaged out

$$= \sum_{y_2 \cdots y_{k-1} \in \{0,1\}^{k-2}} P(Y_k = 1, y_2 \cdots y_{k-1}, Y_1 = 0 | Y_0 = 1) + \sum_{y_2 \cdots y_{k-1} \in \{0,1\}^{k-2}} P(Y_k = 1, y_2 \cdots y_{k-1}, Y_1 = 1 | Y_0 = 1)$$

Split up the sum into $Y_1 = 1$ and $Y_1 = 0$

$$= \sum_{y_2 \cdots y_{k-1} \in \{0,1\}^{k-2}} P(Y_k = 1, y_2 \cdots y_{k-1}, Y_1 = 0 | Y_0 = 1)$$

$$
\begin{aligned}
&+ \sum_{y_2\cdots y_{k-1}\in\{0,1\}^{k-2}} P(Y_k=1, y_2\cdots y_{k-1}|Y_1=1)P(Y_1=1|Y_0=1) && \text{Using the independence assumption}\\
&= \sum_{y_2\cdots y_{k-1}\in\{0,1\}^{k-2}} P(Y_k=1, y_2\cdots y_{k-1}, Y_1=0|Y_0=1)\\
&\quad + \underbrace{P(Y_{k-1}=1|Y_0=1)P(Y_1=1|Y_0=1)}_{=\gamma_{k-1}\beta_1}\\
&= \sum_{y_3\cdots y_{k-1}\in\{0,1\}^{k-3}} P(Y_k=1, y_3\cdots y_{k-1}, Y_2=0, Y_1=0|Y_0=1) && \text{Next, split } Y_2=1 \text{ and } Y_2=0\\
&\quad + \sum_{y_3\cdots y_{k-1}\in\{0,1\}^{k-3}} P(Y_k=1, y_2\cdots y_{k-1}, Y_2=1, Y_1=0|Y_0=1)\\
&\quad + \gamma_{k-1}\beta_1\\
&= \sum_{y_3\cdots y_{k-1}\in\{0,1\}^{k-3}} P(Y_k=1, y_3\cdots y_{k-1}, Y_2=0, Y_1=0|Y_0=1)\\
&\quad + \underbrace{P(Y_{k-2}=1|Y_0=1)P(Y_2=1, Y_1=0|Y_0=1)}_{=\gamma_{k-2}\beta_2} && \text{Using the independence assumption}\\
&\quad + \gamma_{k-1}\beta_1\\
& && \text{Proceed until } Y_{k-1}\\
&= \sum_{y_{k-1}\in\{0,1\}} P(Y_k=1, y_{k-1}, Y_{k-2}=0\cdots Y_1=0|Y_0=1)\\
&\quad + \sum_{i=1}^{k-2}\gamma_{k-i}\beta_i\\
&= \underbrace{P(Y_k=1, Y_{k-1}=0, Y_{k-2}=0\cdots Y_1=0|Y_0=1)}_{=\beta_k} && \text{Split } Y_{k-1}=0 \text{ and } Y_{k-1}=1\\
&\quad + \underbrace{P(Y_1=1|Y_0=1)P(Y_{k-1}=1, Y_{k-2}=0\cdots Y_1=0|Y_0=1)}_{=\gamma_1\beta_{k-1}} && \text{Using the independence assumption}\\
&\quad + \sum_{i=1}^{k-2}\gamma_{k-i}\beta_i\\
\gamma_k &= \beta_k + \sum_{i=1}^{k-1}\gamma_{k-i}\beta_i.
\end{aligned}
$$

Finally, solving for β_k establishes Equation (9) and finishes the proof. □

4.2 Overlapping hits with respect to both DNA strands

In this section, we state the approximation of the *principal overlapping hit probabilities* for the case that both DNA strands are scanned for motif hits.

The approach for obtaining the *principal overlapping hit probabilities* $\{\beta_k\}_{1\leq k<M}$, $\{\beta_{3',k}\}_{0\leq k<M}$ and $\{\beta_{5',k}\}_{1\leq k<M}$ is similar to the approach presented in the previous section. The main difference is that all intermediate hits on both strands need to be considered in $\{\gamma_k\}_{1\leq k<M}$, $\{\gamma_{3',k}\}_{0\leq k<M}$ and $\{\gamma_{5',k}\}_{1\leq k<M}$.

We consider the ordered sequence of events $Y_1Y_1'Y_2Y_2'Y_3Y_3'\cdots$ from left to right and assume that relative to that ordering, events that are separated by a hit (e.g. $Y_l = 1$ or $Y_l' = 1$) are rendered independent of one another.

Accordingly, we identify the following base cases

$$\beta_{3',0} := \gamma_{3',0} = P(Y_0' = 1|Y_0 = 1) \tag{10}$$

$$\beta_{5',1} := \gamma_{5',1} = P(Y_1 = 1|Y_0' = 1) \tag{11}$$

for which there exist no intermediate events, respectively.

Theorem 2. *Assuming that events that are separated by a hit (e.g. $Y_i = 1$ or Y_i') are independent in the sequence $Y_1Y_1'Y_2Y_2'Y_3Y_3'\cdots$ we can express the principal overlapping hit probabilities as*

$$\beta_k = \gamma_k - \sum_{j=1}^{k-1}\gamma_{k-j}\beta_j - \sum_{j=0}^{k-1}\gamma_{3',k-j}\beta_{5',j} \tag{12}$$

$$\beta_{3',k} = \gamma_{3',k} - \sum_{j=1}^{k-1}\gamma_{k-j}\beta_{3',j} - \sum_{j=1}^{k}\gamma_{3',k-j}\beta_j \tag{13}$$

$$\beta_{5',k} = \gamma_{5',k} - \sum_{j=1}^{k-1}\gamma_{k-j}\beta_{5',j} - \sum_{j=1}^{k-1}\gamma_{5',k-j}\beta_j \tag{14}$$

Proof. The proof is a analogous of the proof for Theorem (1). □

5 Adaptation of the original compound Poisson model

We reimplemented the compound Poisson approximation according to Pape *et al.*, 2008 with a few changes, in order to ensure a fair comparison with the improved version. In this section, we briefly summarize the changes to the original version.

First, while the original version supports only order-0 background models, we utilized the afore-mentioned algorithm for computing the two-dimensional distribution of the scores which assumes a higher-order background model. This enables us to compare the original and the improved model across different background model orders.

Second, while in the original version two kinds of overlapping hit probabilities were introduced, namely γ_k and $\gamma'_k = \gamma_{3',k}$, we suggested a third case which is represented by $\gamma_{5',k}$, because $\gamma_{5',k}$ and $\gamma_{3',k}$ are generally not symmetric (see main text). We adapted the original model to include $\gamma_{5',k}$ in the derivation of the clump size probabilities.

Third, in contrast to the original model where a 1-clump is always contained on the forward strand hit, we allowed the 1-clump to either contain a forward strand hit or a reverse strand hit.

Table S1: *Performance comparison for e47: Difference between $P_E(X)$ and the analytic models according to $d_{5\%}$ (see main text). d denotes the order of the background model, α the false positive probability of obtaining a TFBS and seqlen denotes the sequence length.*

d	α	seqlen	$d_{5\%}(P_E, P_{CP}^N)$	$d_{5\%}(P_E, P_{CP}^P)$	$d_{5\%}(P_E, P_{Bin})$
1	10^{-2}	1000	0.0385	0.0431	0.0109
0	10^{-3}	10000	0.00568	0.00579	0.00538
1	10^{-3}	10000	0.00205	0.00216	0.00997
2	10^{-3}	10000	0.00756	0.00765	0.00423

Table S2: *Performance comparison for the Palindromic motif: Difference between $P_E(X)$ and the analytic models according to $d_{5\%}$ (see main text). d denotes the order of the background model, α the false positive probability of obtaining a TFBS and seqlen denotes the sequence length.*

d	α	seqlen	$d_{5\%}(P_E, P_{CP}^N)$	$d_{5\%}(P_E, P_{CP}^P)$	$d_{5\%}(P_E, P_{Bin})$
1	10^{-2}	1000	0.0199	0.0247	0.074
0	10^{-3}	10000	0.00138	0.00272	0.0811
1	10^{-3}	10000	0.00205	0.00387	0.0721
2	10^{-3}	10000	0.00312	0.00537	0.0762

References

Beckstette, M., Homann, R., Giegerich, R., and Kurtz, S. (2006). Fast index based algorithms and software for matching position specific scoring matrices. *BMC bioinformatics*, **7**(1), 389.

Grant, C. E., Bailey, T. L., and Noble, W. S. (2011). Fimo: scanning for occurrences of a given motif. *Bioinformatics*, **27**(7), 1017–1018.

Karlin, S. and Taylor, H. E. (1981). *A second course in stochastic processes*. Elsevier.

Pape, U. J., Rahmann, S., Sun, F., and Vingron, M. (2008). Compound poisson approximation of the number of occurrences of a position frequency matrix (pfm) on both strands. *Journal of Computational Biology*, **15**(6), 547–564.

Rahmann, S., Müller, T., and Vingron, M. (2003). On the power of profiles for transcription factor binding site detection. *Statistical Applications in Genetics and Molecular Biology*, **2**(1).

Reinert, G., Schbath, S., and Waterman, M. S. (2000). Probabilistic and statistical properties of words: an overview. *Journal of Computational Biology*, **7**(1-2), 1–46.

Table S3: *Performance comparison for the repeat-like motif: Difference between $P_E(X)$ and the analytic models according to $d_{5\%}$ (see main text). d denotes the order of the background model, α the false positive probability of obtaining a TFBS and seqlen denotes the sequence length.*

d	α	seqlen	$d_{5\%}(P_E, P_{CP}^N)$	$d_{5\%}(P_E, P_{CP}^P)$	$d_{5\%}(P_E, P_{Bin})$
1	10^{-2}	1000	0.0111	0.0823	0.0558
0	10^{-3}	10000	0.00355	0.0711	0.0606
1	10^{-3}	10000	0.00506	0.0749	0.0526
2	10^{-3}	10000	0.00361	0.0759	0.0538

Table S4: *Clump size distribution: Total variance between the empirical clump size distribution and the approximated clump size distributions.*

Motif	α	$d(P_E, P_{Clump}^N)$	$d(P_E, P_{Clump}^P)$
E47	10^{-3}	0.00283	0.00174
Pal	10^{-3}	0.0389	0.0215
Rep	10^{-3}	0.0517	0.666

Thomas-Chollier, M., Sand, O., Turatsinze, J.-V., Defrance, M., Vervisch, E., Brohée, S., van Helden, J., *et al.* (2008). Rsat: regulatory sequence analysis tools. *Nucleic acids research*, **36**(suppl 2), W119–W127.

Touzet, H., Varré, J.-S., *et al.* (2007). Efficient and accurate p-value computation for position weight matrices. *Algorithms Mol Biol*, **2**(1510.1186), 1748–7188.

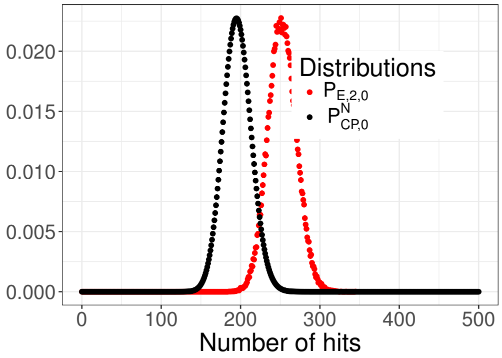

(a) $d = 2$

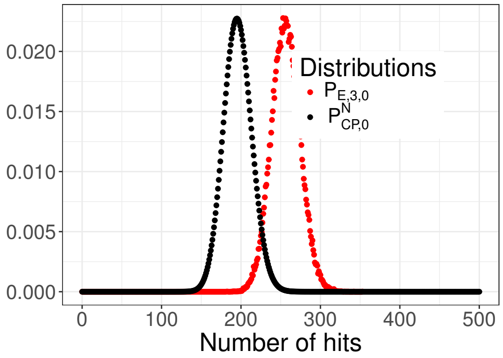

(b) $d = 3$

Figure S1: *A mismatch of the background model order affects the motif hit count distribution. Panel a) and b) illustrate the discrepancy the distributions due to assuming an order-*0 *background when the sequences as actually sampled from an order-*2 *and an order-*3 *model, respectively. (Black) compound Poisson approximation assuming an order-*0 *background. (Red) empirical distribution that is sampled from an order-d background model (but still uses the order-*0 *background to determine the scores to make the scores comparable).*

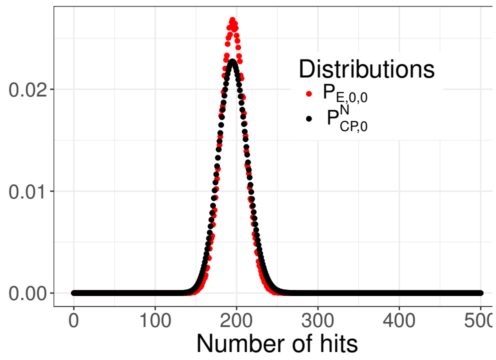

(a) $d = 0$

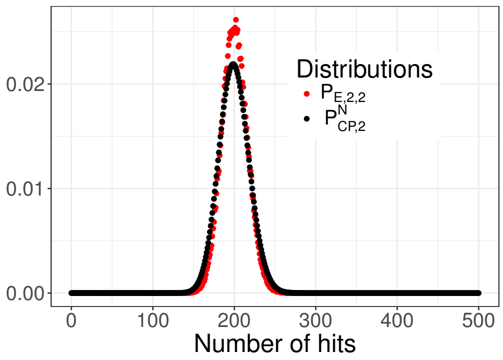

(b) $d = 2$

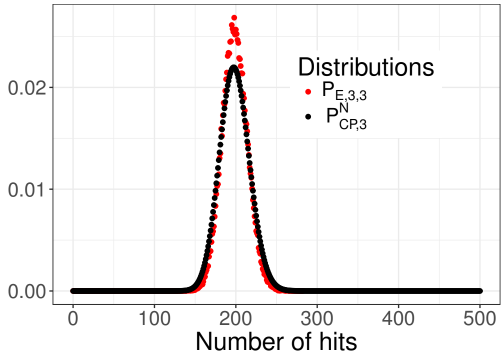

(c) $d = 3$

Figure S2: *Matched background orders lead to similar motif hit count distributions. Panel a)-c) illustrate the compound Poisson approximation (black) and the empirical distribution (red). In each panel, both distributions consider matched background model orders 0, 2 and 3, respectively.*

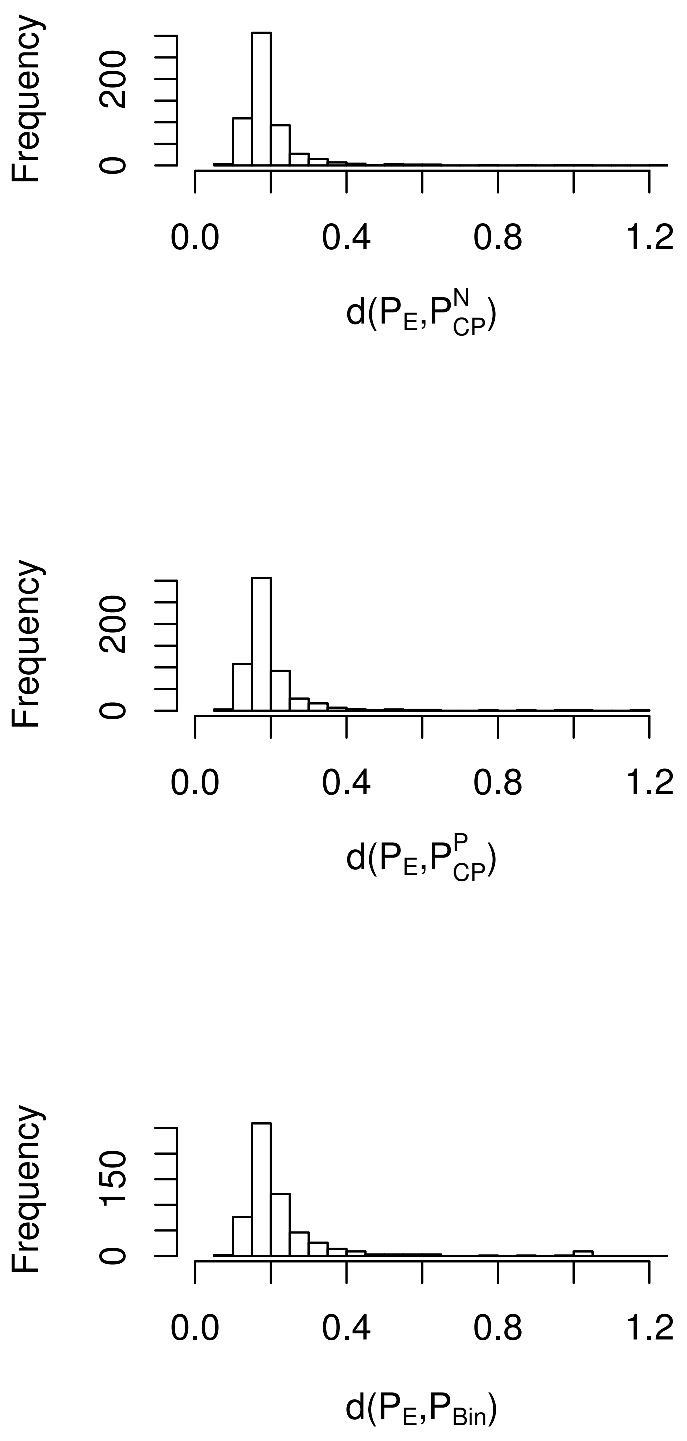

Figure S3: *Histogram of total variation across all Jaspar motifs between the reference P_E and P_{CP}^N, P_{CP}^P and P_{Bin}, respectively.*

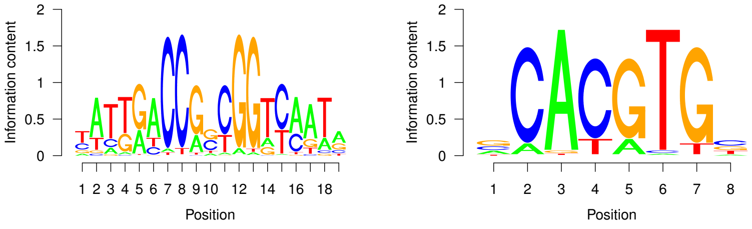

(a) *LFY* **(b)** *MYC3*

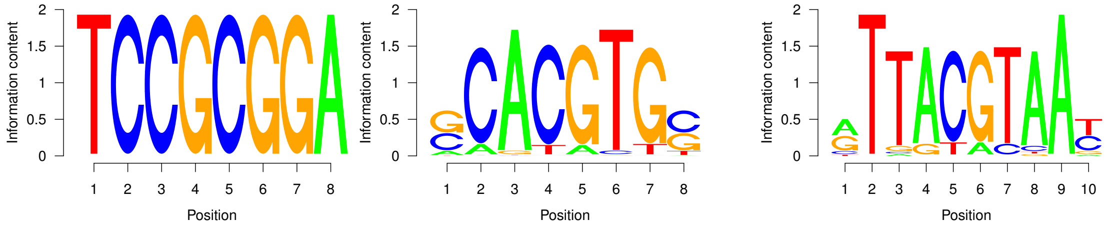

(c) *PDR3* **(d)** *PHO4* **(e)** *gt*

Figure S4: *Examples of Jaspar motifs for which* P_{CP}^{N} *improves over* P_{Bin}.

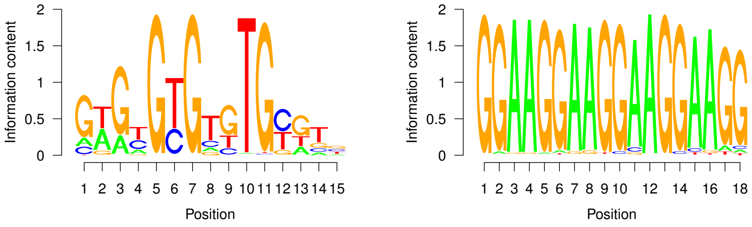

(a) *DAF-12* **(b)** *EWSR1-FLI1*

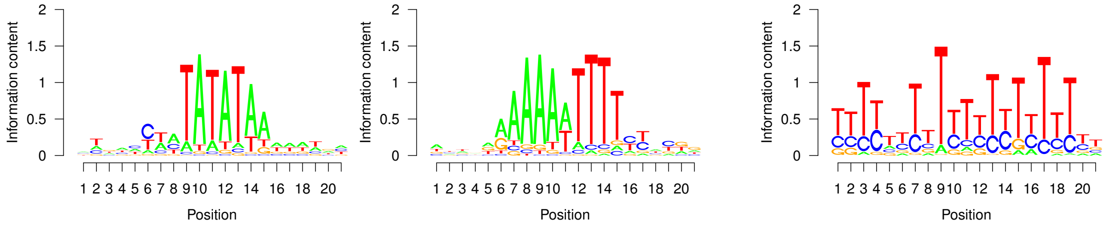

(c) *NHP6A and NHP6B* **(d)** *HMGA2* **(e)** *K2B*

Figure S5: *Examples of Jaspar motifs for which* P_{CP}^{N} *improves over* P_{CP}^{P}.

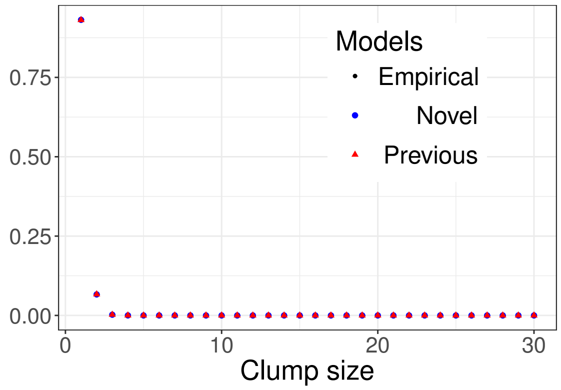

(a) *E47*

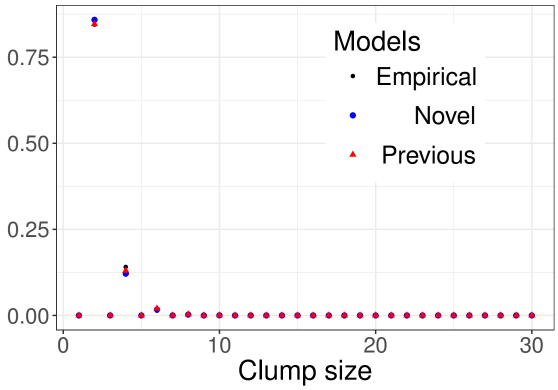

(b) *Palindrome*

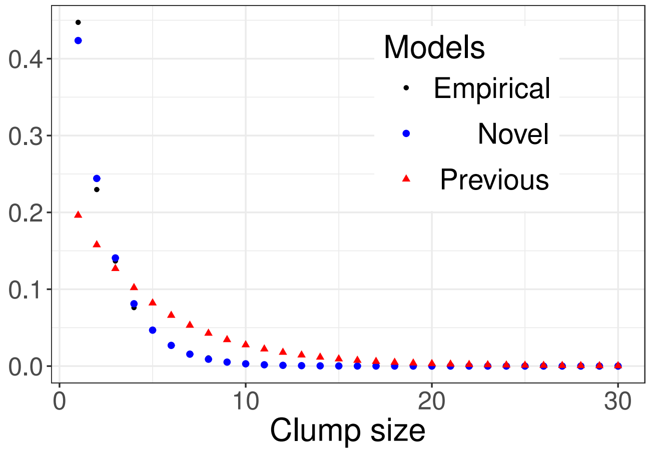

(c) *Rep. motif*

Figure S6: *Clump size distribution: Panel a)-c) show the clump size distributions for E47, a Palindrome and a repeat-like motif using an order-*1 *background model. In each subfigure, the empirical clump size distribution (black) the novel clump size approximation (blue) and the previous clump size approximation (red) are depicted.*
